# Estimated Demand for US Hospital Inpatient and Intensive Care Unit Beds for Patients With COVID-19 Based on Comparisons With Wuhan and Guangzhou, China

**DOI:** 10.1001/jamanetworkopen.2020.8297

**Published:** 2020-05-06

**Authors:** Ruoran Li, Caitlin Rivers, Qi Tan, Megan B. Murray, Eric Toner, Marc Lipsitch

**Affiliations:** 1 Harvard T.H. Chan School of Public Health, Center for Communicable Disease Dynamics, Department of Epidemiology, Boston, Massachusetts; 2 Johns Hopkins Bloomberg School of Public Health, Johns Hopkins Center for Health Security and the Department of Environmental Health and Engineering, Baltimore, Maryland; 3 Department of Global Health and Social Medicine, Harvard Medical School, Boston, Massachusetts; 4 Department of Respiratory and Critical Care Medicine, The First Affiliated Hospital of Nanjing Medical University, Nanjing Medical University, Nanjing, China

## Abstract

**Importance:**

Sustained spread of severe acute respiratory syndrome coronavirus 2 (SARS-CoV-2) has happened in major US cities. Capacity needs in cities in China could inform the planning of local health care resources.

**Objectives:**

To describe and compare the intensive care unit (ICU) and inpatient bed needs for patients with coronavirus disease 2019 (COVID-19) in 2 cities in China to estimate the peak ICU bed needs in US cities if an outbreak equivalent to that in Wuhan occurs.

**Design, Setting, and Participants:**

This comparative effectiveness study analyzed the confirmed cases of COVID-19 in Wuhan and Guangzhou, China, from January 10 to February 29, 2020.

**Exposures:**

Timing of disease control measures relative to timing of SARS-CoV-2 community spread.

**Main Outcomes and Measures:**

Number of critical and severe patient–days and peak number of patients with critical and severe illness during the study period.

**Results:**

In Wuhan, strict disease control measures were implemented 6 weeks after sustained local transmission of SARS-CoV-2. Between January 10 and February 29, 2020, patients with COVID-19 accounted for a median (interquartile range) of 429 (25-1143) patients in the ICU and 1521 (111-7202) inpatients with serious illness each day. During the epidemic peak, 19 425 patients (24.5 per 10 000 adults) were hospitalized, 9689 (12.2 per 10 000 adults) were considered in serious condition, and 2087 (2.6 per 10 000 adults) needed critical care per day. In Guangzhou, strict disease control measures were implemented within 1 week of case importation. Between January 24 and February 29, COVID-19 accounted for a median (interquartile range) of 9 (7-12) patients in the ICU and 17 (15-26) inpatients with serious illness each day. During the epidemic peak, 15 patients were in critical condition and 38 were classified as having serious illness. The projected number of prevalent critically ill patients at the peak of a Wuhan-like outbreak in US cities was estimated to range from 2.2 to 4.4 per 10 000 adults, depending on differences in age distribution and comorbidity (ie, hypertension) prevalence.

**Conclusions and Relevance:**

Even after the lockdown of Wuhan on January 23, the number of patients with serious COVID-19 illness continued to rise, exceeding local hospitalization and ICU capacities for at least a month. Plans are urgently needed to mitigate the consequences of COVID-19 outbreaks on the local health care systems in US cities.

## Key Points

**Question:**

What level of hospital capacity is needed to respond to outbreaks of severe coronavirus disease 2019 in US cities, and how is this associated with intervention timing?

**Findings:**

In this comparative effectiveness study, higher inpatient and intensive care unit utilization in Wuhan was compared with lower utilization in Guangzhou, which implemented strict social distancing measures as well as contact tracing and quarantine protocols earlier than Wuhan. The projected number of prevalent critically ill patients at the peak of a Wuhan-like outbreak in US cities was estimated to range from 2.2 to 4.4 per 10 000 adults, depending on differences in age distribution and comorbidity (ie, hypertension) prevalence.

**Meaning:**

The findings of this study suggest that strict disease control strategies should be implemented early to mitigate the demand for inpatient and intensive care unit beds during a coronavirus disease 2019 outbreak.

## Teaser

This comparative effectiveness study describes and compares the intensive care unit and inpatient bed needs for patients with coronavirus disease 2019 (COVID-19) in 2 cities in China to estimate the peak number of intensive care unit beds needed in US cities if an outbreak equivalent to that in Wuhan occurs.

## Introduction

In the 2 months after the first report of 4 cases of atypical pneumonia in Wuhan, China, on December 27, 2019,^1^ the cumulative number of confirmed cases of coronavirus disease 2019 (COVID-19) in the city rose to 49 122, with 2195 deaths by the end of February 2020.^2^ On January 23, Wuhan city shut down in response to the quickly evolving epidemic. All public transportation within, to, and from the city was suspended, and residents were barred from leaving. An estimated 9 million people remained in the city after the lockdown.^3^ Strict social distancing measures were also implemented, including the compulsory wearing of face masks in public.

During the early phase of the response in Wuhan, the number of patients overwhelmed local fever clinics and hospitals designated to receive patients with COVID-19. The media reported a significant shortage of hospital beds, intensive care unit (ICU) beds, and other health care resources. By February 12, more than 18 000 health care workers had been sent to Wuhan from other parts of China to help with the coronavirus response.^4^ A total of 48 hospitals (including 2 new hospitals built specifically for patients with COVID-19) and more than 26 000 inpatient beds were designated for the isolation and treatment of patients with confirmed severe acute respiratory syndrome coronavirus 2 (SARS-CoV-2). Quarantine centers with more than 13 000 total beds were also established to isolate confirmed patients with milder illness. By the end of February, the local government reported that “finally patients don’t need to wait for beds. Now the beds are waiting for patients.”^5^

With human-to-human transmission now established in other countries, mitigating the potential consequences of COVID-19 on local health care systems is a top priority. A clinical study from China^1^ reported that 81% of patients in whom SARS-CoV-2 is detected experience mild disease, 14% experience severe disease, and 5% experience critical disease. However, questions still remain regarding the proportion of asymptomatic patients and the clinical course of the disease, preventing accurate prediction of hospitalization and ICU needs with transmission models.

Here, we describe the ICU and hospitalization needs for COVID-19 in 2 cities in China, ie, Wuhan, the epicenter of China’s outbreak, and Guangzhou, a metropolis that experienced an early importation of cases. As in all cities in China, Guangzhou implemented strict social distancing measures, contact tracing, and quarantine protocols in late January, which resulted in a much smaller outbreak size than in Wuhan. Describing and comparing the resource needs in both cities may create benchmarks to help other large metropolises prepare for potential outbreaks.

## Methods

All data were extracted from publicly available sources with deidentified information. Per the Common Rule, institutional review board review and informed consent were not required for this study because of non–human participant research determination. We adhered to the International Society for Pharmacoeconomics and Outcomes Research (ISPOR) reporting guideline for comparative effectiveness research^6^ in the design and reporting of this study.

We extracted and estimated confirmed COVID-19 case counts for severe and critical cases from Wuhan and Guangzhou from situation updates from Chinese national and local health commissions. We extracted the number of designated COVID-19 beds and hospitalizations from the Wuhan Municipal Health Commission website.

A confirmed COVID-19 case was considered severe if the patient experienced at least 1 of the following: dyspnea, respiratory frequency of at least 30 per minute, blood oxygen saturation of 93% or lower, arterial blood oxygen partial pressure (Pao_2_) to oxygen concentration (FiO_2_) ratio of less than 300 mm Hg, and/or pneumonia showing significant progression of lesions infiltrating more than 50% of the lung field on chest imaging within 24 to 48 hours. A confirmed patient was considered critical if they experienced respiratory failure demanding invasive and/or noninvasive ventilation for respiratory support, experienced septic shock, and/or had multiple organ dysfunction or failure demanding intensive care.^7,8^ These definitions have been more detailed with revisions of the Chinese diagnostic and treatment guidelines. In this study, we used the phrase *patients with serious illness *to describe patients with severe and critical illness collectively. We estimated the number of prevalent severe and critical cases cross-sectionally per day, allowing for the fact that patients could move in and out of these categories during the course of the disease.

We extracted Wuhan city and Hubei province COVID-19 data between January 10 and February 29, including the numbers of confirmed cases, new recovered cases, new deaths, severe cases, critical cases, serious cases (ie, sum of severe and critical cases), cumulative recovered cases, cumulative deaths, cumulative confirmed cases, and currently confirmed cases (ie, cumulative confirmed cases − deaths − recovered cases). If official sources did not have data for variables on some dates, we calculated the number of cases based on the relationships between variables. Because Wuhan did not systematically report the number of severe and critical cases, we estimated these numbers by assuming that the proportion of serious and critical cases out of all currently confirmed cases was the same in Wuhan as in the rest of Hubei. For dates when it was not possible to estimate the severe and critical case counts using these methods (ie, January 18, 25, and 27), we assumed the number of severe and critical cases were the same as what was reported the previous day. For Guangzhou, we extracted the city’s case count on the number of confirmed, severe, clinical, and recovered cases and deaths for each day between January 24 and February 29.

### Statistical Analysis

We summed the total patient-days under critical and/or severe condition to estimate the total ICU-days and serious inpatient–days. We plotted the raw number of patients in critical and severe condition and patients hospitalized on each day for Wuhan and Guangzhou and estimated the proportion of hospitalizations and ICU admissions per 10 000 adults based on the assumption that there were 9 million people present in Wuhan during the lockdown,^3^ of whom 88.16% were aged 15 years or older,^9^ and 14.9 million people present in Guangzhou, of whom 82.82% were aged 15 years or above.^10^

We then projected the number of patients who would have severe and critical COVID-19 at the peak of a Wuhan-like outbreak in the 30 most populous US cities by assuming that the associations of age and comorbidity with patient outcomes would be the same as their association with COVID-19 mortality, as derived from case reports from China until February 11.^11^ Specifically, we estimated the stratum-specific critical care rate in Wuhan by assuming that the risk factor for being in critical care was the same as that for death (ie, for adults age 65 years or over, rate ratio, 7.2; for adults with hypertention, rate ratio, 6.9).^11^ We estimated the probability of being in critical condition at the peak of the epidemic in each age and hypertension stratum using the COVID-19 mortality rate ratios for age and hypertension^11^ and the proportion of the Wuhan population in each stratum. The hypertension prevalence in adults in Wuhan was estimated as 25.7%,^12^ and the proportion of the population aged 65 years or older was estimated as 14.1%.^13^ We applied these stratum-specific critical care rates to population structures in US cities based on crude hypertension prevalence in adults in 2017^14^ and the proportion of the adult population aged 65 years and older in these cities.^15^ The data on US ICU beds,^16^ empty ICU beds,^17^ inpatient beds,^18^ and population structure^19^ were used to estimate inpatient bed capacity per 10 000 adults. We used *Wuhan-like outbreak* to describe an outbreak in a large metropolis where minimal disease control measures were implemented during the first 2 months of community spread of SARS-CoV-2, followed by city-level lockdown measures to suppress transmission. All analyses were conducted in R version 3.6.3 (R Project for Statistical Computing), and no prespecified level of statistical significance was set.

## Results

**Figure 1.**
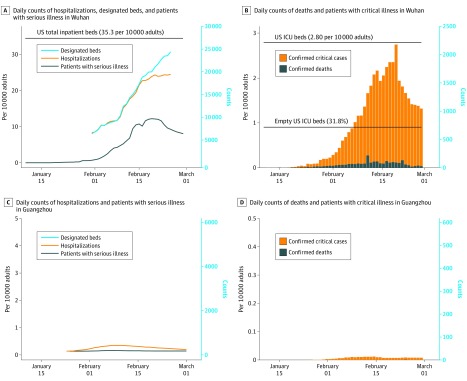
Burden of Serious Coronavirus Disease 2019 in Wuhan and Guangzhou, China Wuhan city locked down on January 23, with a cumulative 495 confirmed cases and 23 deaths among patients with coronavirus disease 2019. Guangzhou initiated level 1 public health response on the same date, with a cumulative 7 confirmed cases and 0 deaths. ICU indicates intensive care unit.

In Wuhan, COVID-19 accounted for a total of 32 486 ICU-days and 176 136 serious inpatient–days between January 10 and February 29 (Figure 1A and Figure 1B), with a median (interquartile range) of 429 (25-1143) patients in the ICU and 1521 (111-7202) inpatients with serious illness each day during that 51-day period. During the peak of the epidemic from mid to late February, a maximum of 19 425 patients (24.5 per 10 000 adults) were hospitalized, 9689 patients (12.2 per 10 000 adults) were considered in serious condition, and 2087 patients (2.6 per 10 000 adults) needed critical care per day.

In Guangzhou, COVID-19 accounted for a total of 318 ICU-days and 724 serious inpatient–days between January 24 and February 29 (Figure 1C and Figure 1D), with a median (interquartile range) of 9 (7-12) patients in the ICU and 17 (15-26) inpatients with serious illness each day during that 37-day period. During the peak of the epidemic (early February), 15 patients were in critical condition, while 38 were hospitalized and classified as serious. Unlike Wuhan, where patients with mild COVID-19 disease were isolated in quarantine centers and not in designated hospitals, all confirmed patients in Guangzhou were hospitalized until recovery. The maximum number of hospitalizations in Guangzhou on any day was 271 patients.

**Figure 2.**
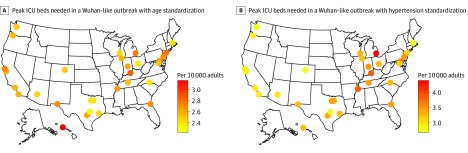
Estimated Number of Critically Ill Patients at the Peak of a Wuhan-Like Outbreak in US Cities per 10 000 Adults In Wuhan, 2.6 individuals per 10 000 adults were critically ill at the peak of the COVID-19 epidemic, with a crude hypertension prevalence of 25.7% among adults (rate ratio for critical illness, 6.9) and 15.9% of adults aged 65 years or older (rate ratio for critical illness, 7.2). ICU indicates intensive care unit.

At the peak of the epidemic, we estimated the critical care risk among adults younger than 65 years to be 1.2 patients per 10 000 adults; among adults aged 65 years or older, 8.0 patients per 10 000 adults; among adults without hypertension, 1.3 patients per 10 000 adults; and among adults with hypertension, 9.5 patients per 10 000 adults. In the 30 most populous cities in the US, 11.0% to 22.5% adults are aged 65 years or older^15^ and the crude hypertension prevalence ranges from 22.0% to 46.9%.^14^ The projected number of prevalent critically ill patients at the peak of a Wuhan-like outbreak in US cities ranged from 2.2 to 3.2 patients per 10 000 adults, when the difference in age distribution was taken into account (Figure 2A) and from 2.8 to 4.4 patients per 10 000 adults when the differences in hypertension prevalence was taken into account (Figure 2B).

## Discussion

Even after the lockdown of Wuhan on January 23, the number of patients with serious COVID-19 cases continued to rise, exceeding local hospitalization and ICU capacities for at least a month. During the peak of the Wuhan epidemic in February, nearly 20 000 patients with COVID-19 were hospitalized simultaneously, with 10 000 in severe or critical condition. If a Wuhan-like outbreak were to take place in a US city, even with social distancing and contact tracing protocols as strict as the Wuhan lockdown, hospitalization and ICU needs from COVID-19 patients alone may exceed current capacity. The need for health care resources may be higher in some US cities, where there is a higher prevalence of vulnerable populations (ie, older age and comorbidity) than in Wuhan.

Exceeding health care capacity may increase the community spread of SARS-CoV-2. In Wuhan, home isolation and quarantine were used in the early phase of the epidemic to alleviate the demand for health care resources. However, because of the exponential increase in the number of patients who developed serious illness but could not be hospitalized owing to capped capacity, secondary transmission in the community continued as patients and their household contacts moved between hospitals seeking care.

Exceeding health care capacity may also lead to decreased quality of care, such as not being able to access a ventilator, which would lead to an increased case-fatality ratio. By the end of February, Wuhan’s case-fatality ratio was 4.5%; it was 3.2% for the rest of Hubei province and 0.8% for the rest of China, where health care capacity was not exceeded because of strong social distancing and contact quarantine measures in the early phase of the epidemic (such as Guangzhou).^20^ A contributing factor to the lower case-fatality ratio in the rest of China may be higher case ascertainment than in Wuhan during the early phase of the epidemic.

In both Wuhan and Guangzhou, the lockdowns did not lead to immediate downturns in demand for hospitalization or the number of serious cases; rather, the peak occurred approximately a month after the lockdown in Wuhan and 2 weeks after the lockdown in Guangzhou. This delay reflects the potentially long time from infection to severe and critical condition, as many patients with COVID-19 who eventually require ICU care present initially with only mild symptoms,^21^ and an even longer time to discharge or death,^22^ resulting in the accumulation of hospitalized cases long after downturns in community spread. In Wuhan, the longer delay may also reflect ongoing transmission after the lockdown, as described earlier, which itself resulted from overloading the health care system.

Historical evidence has shown that, in 1918, the US cities that imposed nonpharmaceutical interventions early in the influenza epidemic course and maintained these interventions during a long period had lower epidemic peaks and fewer total cases than those that waited.^23,24^ Although it included only 2 cities, our comparison of Wuhan with Guangzhou dramatically illustrates the same association of early intervention with lower total number of cases and epidemic peaks. Of course, the future course of these epidemics and others around the world depends on the ability to maintain burdensome control measures over an extended period.

In several locations with high-performing health care systems where SARS-CoV-2 transmission had been established earlier (ie, Hong Kong, Singapore, and Japan), both supplies of personal protective equipment in hospitals and the availability of health care services have been problematic for COVID-19 care, and in all locations, ICU bed capacity is limited.^25^ Combined with other evidence about the consequences of early and intense interventions to control viral spread,^23,24,26^ the comparison with the Guangzhou situation dramatically illustrates that early intervention leads to lower epidemic sizes and peaks and that plans are urgently needed to mitigate the consequences of COVID-19 outbreaks on the health care systems of US cities.

### Limitations

This study has several limitations. We relied on officially reported statistics, which may not represent the change of actual case counts over time but rather reflect the capacity of testing and hospitalization. Thus, the number of serious cases and hospitalizations in Wuhan is not reflective of actual need but rather of the maximum capacity of the system of diagnosis and treatment. Therefore, we are more confident regarding the hospitalization and serious case counts in Wuhan after mid-February and in Guangzhou, where capacities in diagnosis and treatment were not exceeded according to both official and unofficial sources. Furthermore, we have only accounted for the differences in age and hypertension distribution between Wuhan and US cities, but we did not account for other potential risk factors, such as diabetes, cardiovascular diseases, and chronic respiratory diseases.^11^ Because no mutually adjusted associations of these risk factors with COVID-19 serious illness or death were available at the time of our analysis and because cardiometabolic risk factors likely coexist in the same population, we used hypertension adjustment as a proxy for adjusting other known comorbidities.

In addition, our projection of the ICU bed needs in US cities did not consider scenarios in which local transmission differs from that of Wuhan. The contact rate in Wuhan during the early phase of the epidemic may have been much higher than what we expect to occur in US cities because of the increased number of social contacts that occurred in Wuhan because of the lunar new year celebrations. If social distancing measures are effectively implemented early in US cities, the growth of the epidemic may be delayed. But it is also possible that US cities may not be able to implement the extreme social distancing measures that were put in place later in Wuhan. We further assumed that both settings had an equal (age- or hypertension-specific) incidence rate of severe and critical COVID-19 cases, but we did not account for differences in contact patterns in vulnerable populations, such as in nursing homes. Therefore, the actual number of hospital and ICU beds that will be needed during the course of a COVID-19 outbreak in a US city is impossible to estimate precisely. Our estimated capacity needs based on a Wuhan-like outbreak could be a benchmark for what health care systems would expect to see during the first 3 months of a local COVID-19 epidemic if the same outbreak control measures were implemented as in Wuhan.

## Conclusions

Even after the lockdown of Wuhan on January 23, 2020, the number of patients with serious COVID-19 cases continued to rise, exceeding local hospitalization and ICU capacities for at least a month. Plans are urgently needed to mitigate the consequences of COVID-19 outbreaks on local health care systems in US cities.
